# High-Sensitivity Determination of K, Ca, Na, and Mg in Salt Mines Samples by Atomic Emission Spectrometry with a Miniaturized Liquid Cathode Glow Discharge

**DOI:** 10.1155/2017/7105831

**Published:** 2017-11-07

**Authors:** Jie Yu, Zhichao Zhang, Quanfang Lu, Duixiong Sun, Shuwen Zhu, Xiaomin Zhang, Xing Wang, Wu Yang

**Affiliations:** ^1^Key Lab of Bioelectrochemistry and Environmental Analysis of Gansu Province, College of Chemistry and Chemical Engineering, Northwest Normal University, Lanzhou 730070, China; ^2^Editorial Department of the University Journal, Northwest Normal University, Lanzhou 730070, China; ^3^Key Lab of Atomic and Molecular Physics & Functional Materials of Gansu Province, College of Physics and Electronic Engineering, Northwest Normal University, Lanzhou 730070, China

## Abstract

An atomic emission spectrometer (AES) based on a novel atmospheric pressure liquid cathode glow discharge (LCGD) as one of the most promising miniaturized excitation sources has been developed, in which the glow discharge is produced between a needle-like Pt anode and the electrolyte (as cathode) overflowing from a quartz capillary. Lower energy consumption (<50 W) and higher excitation efficiency can be realized by point discharge of the needle-like Pt. The miniaturized LCGD seems particularly well suited to rapid and high-sensitivity determination of K, Ca, Na, and Mg in salt mines samples. The optimized analytical conditions of LCGD-AES were pH = 1 with HNO_3_ as electrolyte, 650 V discharge voltage, and 3 mL min^−1^ solution flow rate. The limits of detections (LODs) of K, Ca, Na, and Mg were 0.390, 0.054, 0.048, and 0.032 mg L^−1^, respectively. Measurement results of the LCGD-AES are in good agreement with the comparison value obtained by inductively coupled plasma (ICP) and ion chromatography (IC). All results suggested that the developed portable analytical instrument can be used for on-site and real-time monitoring of metal elements in field with further improvement.

## 1. Introduction

Salt mines represent a natural mineral resource because they contain many useful components, such as K, Ca, Na, and Mg. Many salt mines are exploited for raw materials used in industry, agriculture, and medicine [1]. However, before the mining and processing, we often need to determine the metal contents. Only in this way will the salt mines enjoy more potential applications. Traditional analysis tools such as AAS, ICP-AES, and ICP-MS are widely used for the determination of metal contents in a variety of real samples. However, these tools are generally confined to the laboratory and required high temperatures, high vacuum, high power input, or even inert/special gases [2, 3]. In addition, it is difficult for ICP to introduce high-salinity solutions, because the salinity load may cause signal suppression, spectral interferences, plasma instability, and even nebulizers blocking [4]. These shortcomings limit their use only in laboratory and do not meet the requirements for the field deployment or real-time monitoring [4, 5]. Therefore, it is necessary to develop a simple, convenient, and portable analytical technique.

Over the past two decades, electrolyte cathode atmospheric glow discharge (ELCAD) has received a rapid development [2, 3, 6, 7]. It is considered as one of the most promising alternative miniaturized excitation sources with potential advantages of commercial and analytical success of the plasma sources (i.e., ICP), because it is more compact and is a portable instrument with lower energy consumption (<75 W) and needs no special sample introduction system like a spray chamber and a nebulizer to transport the analytes to the analytical zone [2, 3, 7]. Also, for ELCAD, the analyte is directly introduced to the plasma without the use of nebulizer, which results in reduced memory effects and avoids the problem of deposits blocking the system [4]. Therefore, it is well suited to the field deployment and on-line analysis of multielements in salt mines samples.

The first use of liquid microplasma for the incorporation of the solution as a cathode for the analysis of atomic spectra was performed in 1993 by Cserfalvi et al. [6] who named this source as electrolyte cathode atmospheric glow discharge (ELCAD) [6, 8]. The main device of ELCAD is a very simple atomization/excitation source; that is, the sample solution is used as a cathode, which overflows from the pipette to the reservoir filled with the electrolyte solution and the tip of the pipette is raised by 1–3 mm to make it electrically conductive, and a counter electrode (mostly W or Pt rod) above it (2–4 mm) is the anode [2, 3, 6, 8]. Following this pioneering study, significant variations of the ELCAD design have been developed, including solution cathode glow discharge (SCGD) [9, 10], direct current atmospheric pressure glow discharge (DC-APGD) [11, 12], liquid sampling-atmospheric pressure glow discharge (LS-APGD) [13, 14], liquid electrode plasma atomic emission spectroscopy (LEP-AES) [15, 16], alternating current electrolyte atmospheric liquid discharge (AC-EALD) [17, 18], drop spark discharge (DSD) [19, 20], and liquid electrode chip discharge [21, 22]. All these indicated that miniaturized and/or portable apparatus may be the most fruitful applications of ELCAD. Besides, the detectability, discharge stability, and emission efficiency are also improved [3, 7].

In recent years, closed-type ELCAD has been applied for the analysis of several real samples including water samples [18, 23], human hair and stream sediment [24], honeys [25], tuna fish and aquatic plant [26], titanium dioxide [27], zircaloys [28, 29], colloidal silica [5], and soils and spruce needles [11]. Despite these progresses, to the best of our knowledge, ELCAD type is rarely applied for the determination of metal elements in salt mines samples, perhaps because of its complicated matrix.

Recently, based on the principle of ELCAD, we also successfully developed a novel liquid cathode glow discharge-atomic emission spectrometry (LCGD-AES) for the simultaneous determination of multimetal elements in water samples [30] and ores samples [31], in which the glow discharge is sustained between a needle-like Pt anode and the electrolyte (as cathode) overflowing from a quartz capillary. Compared with conventional ELCAD, the LCGD has several advantages. For example, sealed Pt wire into a quartz tube can form a Pt point discharge, which can improve the excitation efficiency and reduce the energy consumption (<66 W). In addition, the insertion of the quartz capillary into the graphite tube excludes the reservoir of ELCAD. Moreover, several knots in peristaltic pump tubing can increase the stability of discharge plasma [30, 31].

In this work, in order to further evaluate the feasibility of the method, simultaneous determination of K, Ca, Na, and Mg in salt mines samples was carried out by LCGD-AES. The stability of LCGD and effects of operation parameters, such as discharge voltage, solution flow rate, supporting electrolyte, solution pH, and interfering substance on emission intensity, were investigated in detail. Moreover, the measurement results of the LCGD-AES were compared with inductively coupled plasma (ICP) and ion chromatography (IC).

## 2. Experimental

### 2.1. Apparatus

**Figure 1:**
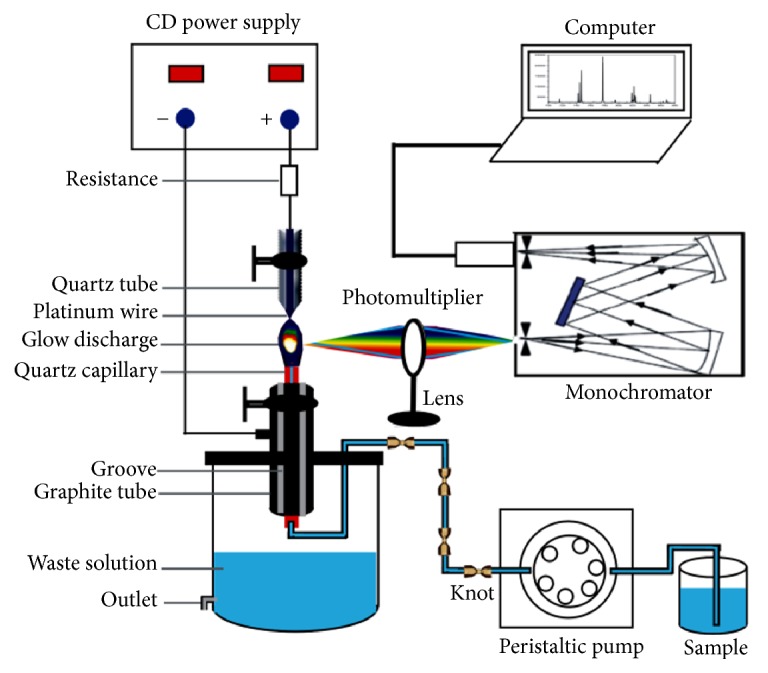
Schematic diagram of the experiment setup.

The schematic diagram of the experimental device for miniaturized LCGD-AES is similar to our previous work [30, 31] and is presented in Figure 1. It contains a DC high voltage power supply, sample introduction, glow discharge system, and spectral detection. The DC high voltage source was a DH 1722-6 power supply (Beijing Dahua Radio Factory, Beijing, China) providing the voltage of 0–1000 V and the current of 0–0.5 A.

The sample solution was pumped into the LCGD system through a quartz capillary (1.0 mm inner diameter and 1.2 mm outer diameter) with the aid of a peristaltic pump (YZ1515X, Beijing Dongnan Yicheng Laboratory Equipment Co., Ltd.). To reduce signal fluctuations of discharge induced by the peristaltic pump, several knots were tied in the peristaltic pump tubing.

The excitation source system consists of two parts: a pointed Pt wire (diameter: 0.5 mm) called the anode was sealed into a conical quartz tube and positioned 1.0 mm above the top of quartz to form a needle-like Pt tip discharge that results in a higher excitation efficiency, while the sample solution was introduced through the quartz capillary and flowed over the top of the capillary into the grooves on the graphite tube, which in turn was served as the liquid cathode. The vertical distance between capillary and pointed Pt wire is 2 mm. The capillary was inserted into a graphite tube (1.2 mm inner diameter and 5.0 mm outer diameter) and protruded from the graphite tube about 2.5 mm. The graphite tube was fixed on the plug of waste reservoir. The solution overflowing from the top of the quartz capillary was flowed into the waste reservoir through many grooves on the graphite tube. The excitation source system was installed on a manual precision translation stage with three orthogonal micrometer screw gauges, which could be controlled precisely in the *x*, *y*, and *z* directions to adjust position of the glow plasma, obtain the maximum signal output, and focus the glow image into the entrance slit of monochromator (Omni-*λ*500, Zolix Instruments Co., Ltd.) with a 1800 grooves/mm holographic grating.

The emission spectrometry of glow discharge was imaged with a quartz lens (diameter: 5 cm; focal length: 10 cm) into the vertical adjustable entrance slit of the monochromator. A PMTH-S1-CR131 photomultiplier (PMT) running at −1000 V was used as the detector. Monochromator control and data acquisition were performed with the ZolixScan Basic V4 based software integral to the Omni-*λ*500. Spectral resolution of the monochromator was 0.05 nm, and integration time was set as 100 ms for each measurement at 0.1 nm intervals.

### 2.2. Reagents and Samples

HNO_3_, HCl, and H_2_SO_4_ were of superior reagent grade and were supplied by Sinopharm Chemical Reagent Co., Ltd. (Ningbo, China). 1000 mg L^−1^ stock standards of K, Ca, Na, and Mg were obtained from the National Research Center for Certified Reference Materials (Beijing, China). Background electrolyte was adjusted to pH = 1 with HNO_3_. Working standard solutions were prepared by appropriate dilution from the corresponding stock solutions and then adjusted to pH = 1.0 with HNO_3_. The pH of the solutions was measured with a pH meter (PHS-3E, INESA, China). Deionized water (18.25 MΩ cm) purified using a water purification system (Sanshen Medical Devices Co., Ltd., Shanghai, China) was used for the preparation of all working solutions.

Four preprocessed salt mines (denoted as A, B, C, and D) were supplied by Qinghai Salt Lake Potash Fertilizer Co., Ltd (Geermu, China). All samples treatment procedures were as follows: each real sample was weighed accurately 0.500 g and then dissolved, transferred into a 100 mL volumetric flask and adjusted to pH = 1.0 with HNO_3_, and then separated by high-speed centrifuge at 10000 r min^−1^ to obtain measured solution samples. After that, the contents of Ca, Na, and Mg in the solution samples were directly determined by LCGD-AES, ICP-AES, and IC. In addition, each solution sample (1 mL) was diluted 100 times with pH = 1.0 HNO_3_ solution to determine the content of K because the concentration of K in solution sample is much higher than that of Ca, Na, and Mg. All data points represent the average values from the 10 successive measurements.

## 3. Results and Discussion

### 3.1. Emission Spectra of the LCGD-AES

**Figure 2:**
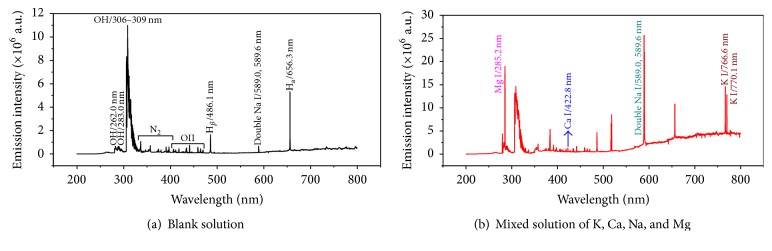
Typical LCGD-AES spectrum of the pH = 1.0 HNO_3_ blank solution (a) and 50 mg L^−1^ K, Ca, Na, and Mg mixture solution adjusted to pH = 1 with HNO_3_ (b) (discharge voltage: 650 V, flow rate: 3 mL min^−1^, interelectrode gap: 2 mm, and capillary diameter: 1 mm).

In order to verify the feasibility of this method, the blank solution (pH = 1.0 HNO_3_) (a) and 50 mg L^−1^ mixed solution of K, Ca, Na, and Mg (adjusted to pH = 1.0 with HNO_3_) (b) were introduced into the LCGD-AES.  Figure 2 shows the typical emission spectra between 200 and 800 nm. As shown in Figure 2(a), the bands in the wavelength from 262.0 to 309.0 nm are attributed to the emission of OH (A^2^Σ + →X^2^Π) [32, 33]. Molecular band spectra of N_2_ ranging from 315 to 406 nm and ascribed to the C^3^Π_u_ → B^3^Π_g_ systems are observed in emission spectra because the discharge is carried out in ambient air. In addition, a series of O II lines are distributed from 410.0 to 470.1 nm, which are produced from water vapor by electron impact. Moreover, the atomic lines of H_*α*_ and H_*β*_ are at 656.3 and 486.1 nm, which come from the electrolyte around the cathode that is bombarded by the high energy electrons [30, 31]. Spectral lines of Na I also appear at 589.0 and 589.6 nm, which suggests that the blank sample still contains a small amount of impurities.

However, when we added K, Ca, Na, and Mg to the blank solution (shown Figure 2(b)), the new lines of K I, Ca I, Na I, and Mg I were observed at 766.5 or 770.1 nm, 422.7 nm, 589.0 nm or 589.6 nm, and 285.2 nm, respectively. As can be seen from Figure 2(b), K I 766.5 nm is stronger than 770.1 nm and Na I 589.0 nm is stronger than 589.6 nm. What is more, all lines are clearly isolated from the blank emission spectra. Therefore, the lines of 766.5, 422.7, 589.0, and 285.2 nm are selected as analytical lines of K I, Ca I, Na I, and Mg I, respectively. All these results indicated that it is viable to use LCGD-AES for simultaneous qualitative identification of K, Ca, Na, and Mg in salt mines.

### 3.2. Optimization of the Experimental Conditions

#### 3.2.1. Effect of Discharge Voltage on Emission Intensity

**Figure 3:**
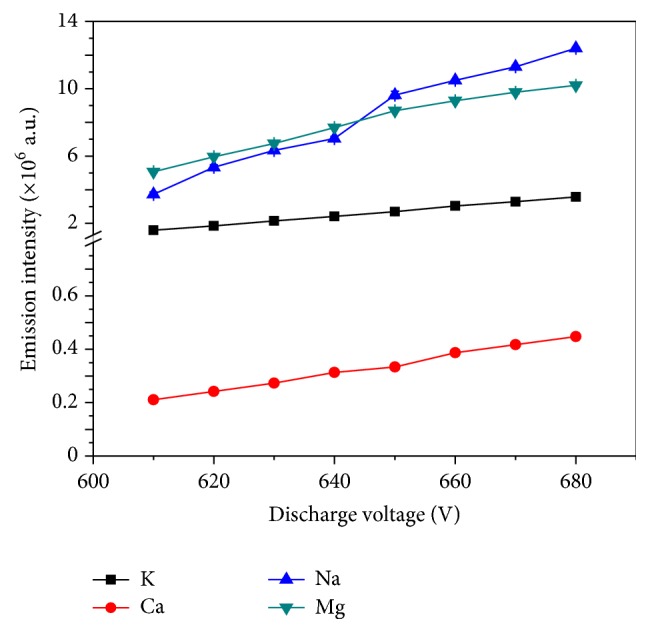
Effect of discharge voltage on emission intensity (concentration: 5 mg L^−1^ adjusted to pH = 1.0 with HNO_3_, flow rate: 3 mL min^−1^, interelectrode gap: 2 mm, and capillary diameter: 1 mm).

With the solution flow rate maintained at 3 mL min^−1^, the effect of discharge voltage on emission intensity was studied. The emission intensity increased significantly with increasing the discharge voltage from 610 to 680 V, as shown in Figure 3. When the discharge voltage is more than 680 V, the Pt cathode turns red and the samples start to boil, which impacts the detection accuracy [30]. A higher voltage also damages the quartz capillary and causes the discharge to become unstable [18]. Therefore, the 650 V is chosen as the optimal discharge voltage in this study.

**Figure 4:**
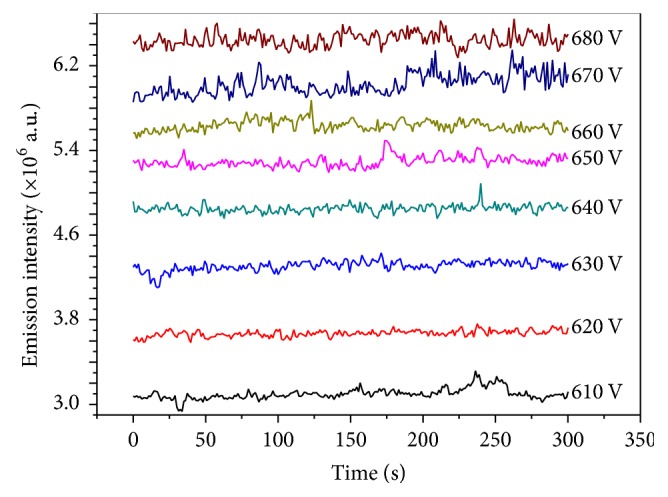
Stability of emission intensity as a function of time of LCGD (electrolytes: 5 mg L^−1^ K solution adjusted to pH = 1.0 with HNO_3_, flow rate: 3 mL min^−1^, interelectrode gap: 2 mm, and capillary diameter: 1 mm).

Under the fixed wavelength at 766.5 nm, the emission intensity of K I was taken as a function of time to test the stability of discharge.  Figure 4 shows the temporal tracing of the emission intensity of 5 mg L^−1^ K solution in different voltages about 5 min after the plasma was stabilized for about 2 min. It is found that the emission intensity is increased with increasing the discharge voltage from 610 to 680 V. However, over 660 V, the increasing voltage will cause fluctuation of the emission intensities because of unstable discharge plasma [30, 31]. Because the emission intensity and stability of discharge are moderate at 650 V, the 650 V discharge voltage was adopted in subsequent studies.

#### 3.2.2. Effect of Solution Flow Rate on Emission Intensity

**Figure 5:**
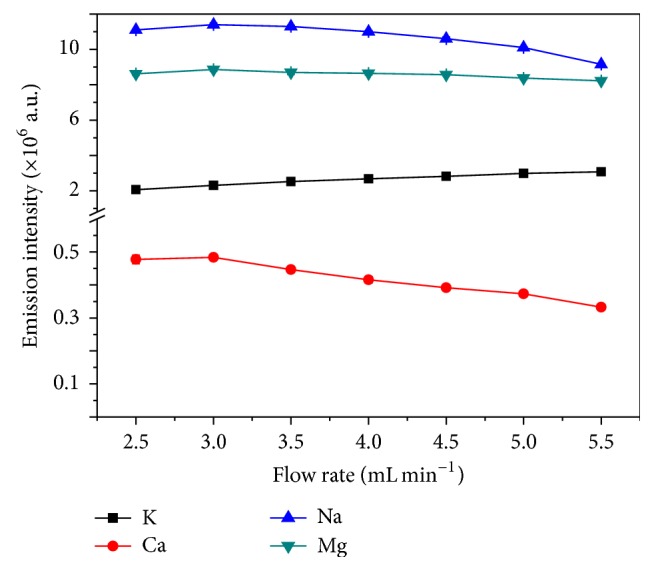
Effect of solution flow rate on emission intensity (electrolytes: 5 mg L^−1^ K, Ca, Na, and Mg solution adjusted to pH = 1.0 with HNO_3_, discharge voltage: 650 V, interelectrode gap: 2 mm, and capillary diameter: 1 mm).

The effect of solution flow rate on emission intensity was also evaluated in the range of 2.5–5.5 mL min^−1^. As shown in Figure 5, the emission intensity of K is increased with flow rate from 2.5 to 5.5 mL min^−1^, but the emission intensities of Ca, Na, and Mg are increased from 2.5 to 3.0 mL min^−1^ and then declined after further increasing the flow rate from 3.0 to 5.5 mL min^−1^. The increase of the emission intensity with the increase of flow rate at lower range may be ascribed to the raised amounts of analytes which entered the discharge [17]. The reduction of emission intensity at higher flow rate may be a consequence of additional water vaporization which may reduce the energy or number of electrons available for exciting the atoms [18]. What is more, increasing the water at higher flow rates might also cool the plasma [34]. Based on these results, 3.0 mL min^−1^ was selected as the optimal solution flow rate.

#### 3.2.3. Effect of the Supporting Electrolyte on Emission Intensity

**Figure 6:**
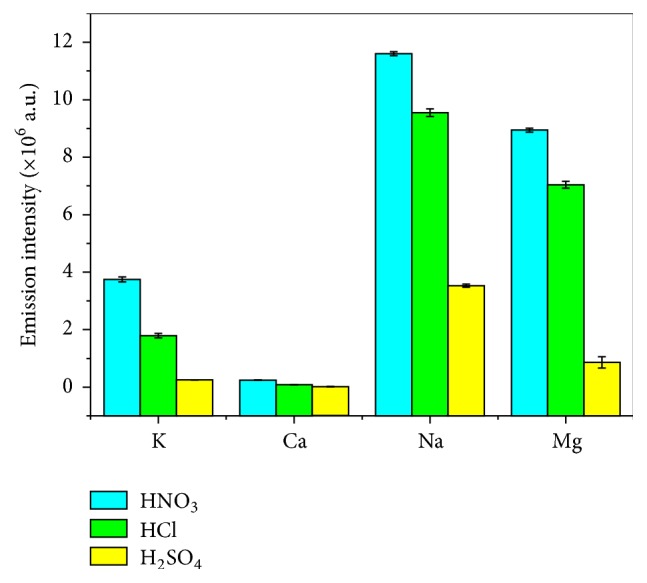
Effect of the supporting electrolyte (electrolytes: 5 mg L^−1^ K, Ca, Na, and Mg solution adjusted to pH = 1.0 by support acid, discharge voltage: 650 V, flow rate: 3 mL min^−1^, interelectrode gap: 2 mm, and capillary diameter: 1 mm).

Mezei et al. [35] found in the ELCAD system that using acids as the electrolyte results in stronger emission than using salts and that the acid anions also affect the emission intensity. Therefore, the effect of the different supporting electrolyte (adjusted to pH = 1.0 with HNO_3_, HCl, and H_2_SO_4_, resp.) on the emission intensity of 5 mg L^−1^ K, Ca, Na, and Mg solutions was investigated. As shown in Figure 6, the net intensities of K, Ca, Na, and Mg are all affected by the acid anions. It was found that the emission intensity follows the order NO^3−^ > Cl^−^ > SO_4_^2−^. It suggested that HNO_3_ exhibits higher emission intensities for K, Ca, Na, and Mg. This result is consistent with what was reported by Mezei et al. [2, 35] and Webb et al. [36]. When the size of the anion is increased, the conductivity of the ions in electrolyte will be reduced, and then the current and power are to become lower [34]. As the size of NO_3_^−^ is close to Cl^−^, the change of emission intensity is not obvious (ionic radii of NO_3_^−^, Cl^−^, and SO_4_^2−^ are 165, 181, and 244 pm, resp.). As the size of SO_4_^2−^ is larger than that of Cl^−^ and NO_3_^−^, the lower emission intensity is observed. What is more, HCl and H_2_SO_4_ are easy to generate precipitation with several metal ions [30, 31]. In addition, HNO_3_ has good sensitivity and chemical compatibility [34]. Therefore, we chose HNO_3_ as supporting electrolyte mediums for subsequent experiments.

#### 3.2.4. The Effect of the Solution pH on Emission Intensity

**Figure 7:**
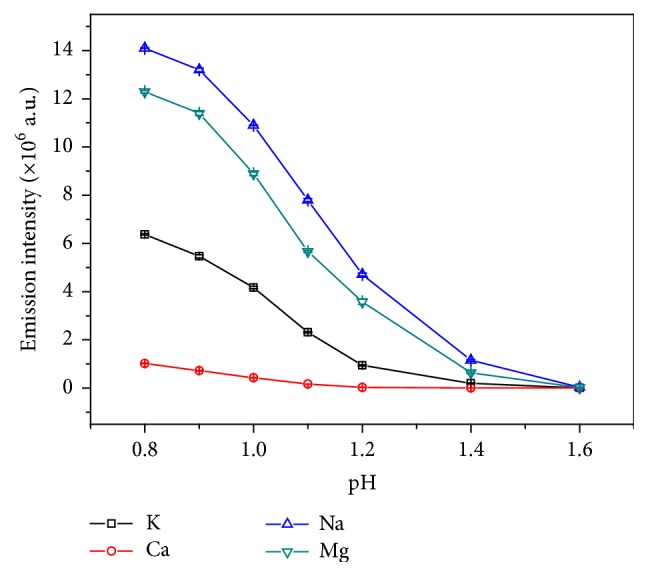
The effect of the solution pH on emission intensity (electrolytes: 5 mg L^−1^ K, Ca, Na, and Mg solution, discharge voltage: 650 V, flow rate: 3 mL min^−1^, interelectrode gap: 2 mm, and capillary diameter: 1 mm).

As we all know, the emission intensity was dependent on the solution pH in ELCAD [6] and AC-EALD [17, 18]. Thus, the effect of the solution pH was also optimized in the present study. It was found that when the pH was lower than 0.8, the glow is very violent and the emission intensity is prone to fluctuation due to the higher conductivity and higher energy. In addition, the Pt and quartz capillary would be destroyed when the pH was below 0.8. However, when the pH is above 1.6, the emission intensity could not be ascertained clearly because of the lower conductivity and weaker glow [30, 31]. Therefore, the effect of solution pH on element emission intensity was studied in the pH range of 0.8–1.6. As shown in Figure 7, the emission intensities were decreased from pH 0.8 to 1.6. By taking into account the emission efficiency, discharge stability, and detectability, we selected pH = 1.0 as the optimum solution pH.

#### 3.2.5. Effect of Interfering Substance on Emission Intensity

**Figure 8:**
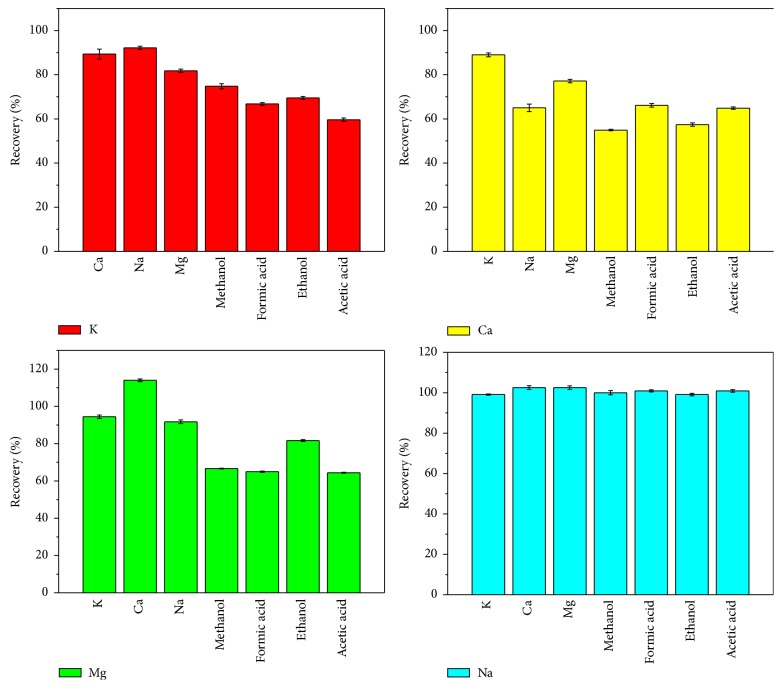
Mutual interference and the influence of organic matter (5 mg L^−1^ K, Ca, Na, and Mg solution adjusted to pH = 1.0 with HNO_3_, discharge voltage: 650 V, flow rate: 3 mL min^−1^, interelectrode gap: 2 mm, and capillary diameter: 1 mm, added to 3% (volume ratio) methanol, ethanol, formic acid, and acetic acid and 50-fold K, Ca, Na, and Mg).

To evaluate the sensitivity of LCGD to the matrix-induced interferences in the analysis of samples, the effects of organic additives (methanol, ethanol, formic acid, and acetic acid) and inorganic metals (K, Ca, Na, and Mg) on the emission intensity were studied, respectively. The potential interfering substances were added separately (3%, volume ratio), methanol, ethanol, formic acid and acetic acid, and 50-fold K, Ca, Na, and Mg, to the single-element working standard solutions at 5 mg L^−1^ with pH = 1.0 HNO_3_.  Figure 8 shows the change of emission intensity with and without foreign substances. It was found that the emission intensities of K and Ca decreased significantly with the addition of foreign substances. However, in the case of the measurements of Na, no remarkable interferences from any foreign substances were observed. It could be observed that the inorganic salt (Ca) could enhance the emission intensity of Mg, but other interfering substances will reduce the emission intensity. Unfortunately, it is difficult to judge the cause of this type of interference. Certainly, this is related to a complex and unexplained mechanism for the release of the analytes from the surface of the flow solution [23].

### 3.3. Analytical Performance

The analytical performance of LCGD-AES was evaluated under optimal operating parameters (supporting electrolytes: adjusted to pH = 1.0 with HNO_3_, discharge voltage: 650 V, flow rate: 3 mL min^−1^, and interelectrode gap: 2 mm). Standard solutions of K, Ca, Na, and Mg ranging from 1 to 10 mg L^−1^ were prepared and established calibration curves. The results showed that all calibration curves have a good linear relationship. The linear equation, LODs, sensitivity, *R*^2^, and RSD are listed in Table 1. It is obvious that *R*^2^ and the RSD ranged from 0.9822 to 0.9981 and from 0.26% to 6.83%, respectively. The LODs of K, Ca, Na, and Mg are 0.390, 0.054, 0.048, and 0.032 mg L^−1^, respectively. The power consumption is 39–47 W. The results suggested that determination of K, Ca, Na, and Mg by using LCGD-AES has high sensitivity and precision and low LOD and power consumption. So it can be employed for quantitative determination of metal elements in salt mines.

A comparison of the LODs obtained by other ELCAD-type [8–14] is listed in Table 2. Obviously, the LODs for LCGD are found to be comparable to those of similar ELCAD systems.

### 3.4. Analysis of the Real Sample

To validate the proposed method, salt mines samples (A, B, C, and D) were applied for the determination of K, Ca, Na, and Mg. The recovery of samples was carried out to verify the accuracy using the standard addition method. In addition, the measurement results of samples obtained by LCGD-AES were also compared with ICP-AES and IC. The measurement results of LCGD, ICP, and IC are listed in Table 3. As shown in Table 3, the measurement results of real samples by using LCGD-AES are in agreement with the comparative values obtained by ICP-AES and IC. Moreover, the recoveries of K, Ca, Na, and Mg by LCGD-AES ranged from 84.05% to 115.94%. All these indicated that the measurement results of LCGD-AES are reliable and accurate.

The statistical* t*-test is widely used for estimating the concordance of an analytical method [37, 38]. The* t*-test method calculates *p* value to find the statistical significance of the test within a specified confidence interval by initially assuming that the means of both groups are identical. Values of *p* < 0.05 (with a confidence interval of 95%) indicate that the groups' means are different. Otherwise *p* attains a value > 0.05, indicating that both groups have identical means [38, 39]. The statistical results between LCGD-AES and IC were listed in Table 4. As can be seen from Table 4, all values of *t* do not go over *t*_95%_ = 2.78 (*t*-test for a confidence level of 95%) except for K measurement in all samples, Ca measurement in sample B, and Mg measurement in samples C and D. In addition, most of* p* values (significant level) are higher than 0.05 (Table 4). This is because K ions concentrations in the samples are very high and dilution can lead to the larger errors. That is to say, some results are in good agreement with the* t*-test for a confidence level of 95%. All results indicated that there is no significant difference between the two methods and suggested that the measurement results using LCGD-AES are roughly reliable and both the techniques are in agreement with each other. Therefore, the developed LCGD-AES has the potential to be applied for the determination of metal in complex salt mines samples.

## 4. Conclusions

The liquid cathode glow discharge-atomic emission spectrometry (LCGD-AES) was successfully applied for measurement of K, Ca, Na, and Mg in salt mines samples. The optimization analytical conditions of LCGD-AES were pH = 1 with HNO_3_ as electrolyte, 650 V voltage, and 3 mL min^−1^ flow rate. The power consumption is below 50 W. *R*^2^ and the RSD ranged from 0.9822 to 0.9981 and from 0.26% to 6.83%, respectively. The LODs of K, Ca, Na, and Mg were 0.390, 0.054, 0.048, 0.032 mg L^−1^, respectively. The recoveries of K, Ca, Na, and Mg by LCGD-AES ranged from 84.05% to 115.95%. The measurement results of LCGD-AES are very consistent with the comparative values of ICP-AES and IC. Compared with ICP-AES, LCGD-AES has some advantages, such as low power consumption, no gas requirement, low cost in setup, and easy operation and design. Moreover, it is easy in achieving real-time and on-line monitoring for samples. All the results suggested that the LCGD-AES is a very promising portable analytical instrument for highly efficient determination of metal elements in salt mines samples.

## Figures and Tables

**Table 1:** Analytical performance of LCGD-AES for K, Ca, Na, and Mg solution.

Analyte	Analytical line (nm)	Power (W)	Calibration equation	*R* ^2^	Sensitivity	LOD (mg L^−1^)	RSD^*∗*^ (%)
K	766.5	40.3–46.8	*I* = 21821 + 233931*C*	0.9981	233931	0.390	0.26
Ca	422.7	41.6–46.2	*I* = 4236 + 81256*C*	0.9973	81256	0.054	6.83
Na	589.0	39.3–44.2	*I* = 3182790 + 1473620*C*	0.9822	1473620	0.048	0.28
Mg	285.2	40.3–46.8	*I* = 589022 + 1846380*C*	0.9918	1846380	0.032	2.83

^*∗*^Standard concentration: 5 mg L^−1^, *n *= 10.

**Table 2:** Comparison of the LODs obtained by LCGD-AES system with other ELCAD-AES systems for the detection of K, Ca, Na, and Mg.

Methods	LOD (mg L^−1^)				Reference
	K	Ca	Na	Mg	
LCGD-AES	0.390	0.054	0.048	0.032	This work
ELCAD	0.200	0.400	0.060	0.800	[8]
Earlier	0.013	0.023	0.0008	0.019	[9]
SCGD	0.0006	0.45	0.0005	0.340	[10]
DC-APGD	0.004	0.36	0.002	0.1	[11]
DC-APGD	0.007	0.150	0.003	0.030	[12]
APGD	0.004	0.09	0.002	0.04	[13]
APGD	0.00085	0.25	0.00023	0.015	[14]

**Table 3:** Measurement results of K, Ca, Na, and Mg in real salt mines by LCGD-AES, ICP, and IC.

Sample	Element	This work (mg g^−1^)	ICP (mg g^−1^)	IC (mg g^−1^)	Recovery^a^ (%)
A	K	599.08 ± 16.55	—	479.20 ± 8.23	107.86
	Ca	0.28 ± 0.04	0.23 ± 0.05	0.31 ± 0.01	84.05
	Na	5.34 ± 1.04	—	3.25 ± 0.03	90.63
	Mg	2.34 ± 0.30	2.66 ± 0.03	2.13 ± 0.05	106.92

B	K	577.93 ± 10.56	—	466.08 ± 5.14	115.94
	Ca	0.33 ± 0.03	0.84 ± 0.05	0.37 ± 0.01	87.16
	Na	4.55 ± 1.10	—	3.60 ± 0.05	98.76
	Mg	2.97 ± 0.13	3.14 ± 0.05	2.84 ± 0.03	103.04

C	K	641.35 ± 21.78	—	488.18 ± 8.56	112.69
	Ca	0.32 ± 0.01	0.31 ± 0.01	0.37 ± 0.01	88.37
	Na	5.06 ± 1.12	—	4.79 ± 0.05	91.83
	Mg	2.71 ± 0.20	3.62 ± 0.07	3.53 ± 0.05	104.02

D	K	641.52 ± 10.42	—	485.82 ± 6.42	109.19
	Ca	0.25 ± 0.04	0.30 ± 0.03	0.28 ± 0.01	94.03
	Na	5.22 ± 1.13	—	4.24 ± 0.05	94.63
	Mg	3.33 ± 0.28	4.50 ± 0.05	4.03 ± 0.03	106.32

^a^Added standard concentration: 5 mg L^−1^, *n* = 10.

**Table 4:** *t*-test and *p* values between the analytical results obtained by LCGD-AES and IC.

Elements	Sample			
	A	B	C	D
	*t* (*p*) values			
K	40.90 (9.7 × 10^−10^)^a^	443.57 (1.9 × 10^−7^)^a^	15.73 (1.3 × 10^−5^)^a^	832.08 (6.4 × 10^−7^)^a^
Ca	1.71 (0.09)	3.22 (0.02)^a^	1.77 (0.62)	1.52 (0.10)
Na	2.28 (0.23)	1.01 (0.15)	0.29 (0.11)	1.03 (0.25)
Mg	1.61 (0.05)	2.22 (0.11)	9.26 (0.01)^a^	5.55 (0.01)^a^

^a^The values of *t*_95%_> 2.78 (with a confidence interval of 95%) and *p* < 0.05 indicated that there was a significant difference between IC and LCGD-AES.

## References

[1] Shadrin N., Zheng M., Oren A. (2015). Past, present and future of saline lakes: research for global sustainable development. *Chinese Journal of Oceanology and Limnology*.

[2] Mezei P., Cserfalvi T. (2007). Electrolyte cathode atmospheric glow discharges for direct solution analysis. *Applied Spectroscopy Reviews*.

[3] He Q., Zhu Z., Hu S. (2014). Flowing and nonflowing liquid electrode discharge microplasma for metal ion detection by optical emission spectrometry. *Applied Spectroscopy Reviews*.

[4] Yang C., Wang L., Zhu Z. (2016). Evaluation of flow injection-solution cathode glow discharge-atomic emission spectrometry for the determination of major elements in brines. *Talanta*.

[5] Wang Z., Schwartz A. J., Ray S. J., Hieftje G. M. (2013). Determination of trace sodium, lithium, magnesium, and potassium impurities in colloidal silica by slurry introduction into an atmospheric-pressure solution-cathode glow discharge and atomic emission spectrometry. *Journal of Analytical Atomic Spectrometry*.

[6] Cserfalvi T., Mezeit P., Apai P. (1993). Emission studies on a glow discharge in atmospheric pressure air using water as a cathode. *Journal of Physics D: Applied Physics*.

[7] Pohl P., Jamroz P., Swiderski K., Dzimitrowicz A., Lesniewicz A. (2017). Critical evaluation of recent achievements in low power glow discharge generated at atmospheric pressure between a flowing liquid cathode and a metallic anode for element analysis by optical emission spectrometry. *TrAC—Trends in Analytical Chemistry*.

[8] Cserfalvi T., Mezei P. (1994). Direct solution analysis by glow discharge: Electrolyte-cathode discharge spectrometry. *Journal of Analytical Atomic Spectrometry*.

[9] Webb M. R., Andrade F. J., Gamez G., McCrindle R., Hieftje G. M. (2005). Spectroscopic and electrical studies of a solution-cathode glow discharge. *Journal of Analytical Atomic Spectrometry*.

[10] Schwartz A. J., Ray S. J., Hieftje G. M. (2016). Evaluation of interference filters for spectral discrimination in solution-cathode glow discharge optical emission spectrometry. *Journal of Analytical Atomic Spectrometry*.

[11] Greda K., Jamróz P., Pohl P. (2013). The improvement of the analytical performance of direct current atmospheric pressure glow discharge generated in contact with the small-sized liquid cathode after the addition of non-ionic surfactants to electrolyte solutions. *Talanta*.

[12] Greda K., Jamroz P., Pohl P. (2013). Comparison of the performance of direct current atmospheric pressure glow microdischarges operated between a small sized flowing liquid cathode and miniature argon or helium flow microjets. *Journal of Analytical Atomic Spectrometry*.

[13] Jamróz P., Pohl P., Zyrnicki W. (2012). An analytical performance of atmospheric pressure glow discharge generated in contact with flowing small size liquid cathode. *Journal of Analytical Atomic Spectrometry*.

[14] Greda K., Jamroz P., Pohl P. (2016). Ultrasonic nebulization atmospheric pressure glow discharge—preliminary study. *Spectrochimica Acta Part B: Atomic Spectroscopy*.

[15] Banno M., Tamiya E., Takamura Y. (2009). Determination of trace amounts of sodium and lithium in zirconium dioxide (ZrO_2_) using liquid electrode plasma optical emission spectrometry. *Analytica Chimica Acta*.

[16] Kitano A., Iiduka A., Yamamoto T., Ukita Y., Tamiya E., Takamura Y. (2011). Highly sensitive elemental analysis for Cd and Pb by liquid electrode plasma atomic emission spectrometry with quartz glass chip and sample flow. *Analytical Chemistry*.

[17] Huang R., Zhu Z., Zheng H., Liu Z., Zhang S., Hu S. (2011). Alternating current driven atmospheric-pressure liquid discharge for the determination of elements with optical emission spectrometry. *Journal of Analytical Atomic Spectrometry*.

[18] Xiao Q., Zhu Z., Zheng H., He H., Huang C., Hu S. (2013). Significant sensitivity improvement of alternating current driven-liquid discharge by using formic acid medium for optical determination of elements. *Talanta*.

[19] Yagov V. V., Korotkov A. S., Zuev B. K., Myasoedov B. F. (1998). Drop-spark discharge: an atomization and excitation source for atomic emission sensors. *Mendeleev Communications*.

[20] Yagov V. V., Getsina M. L., Zuev B. K. (2004). Use of electrolyte jet cathode glow discharges as sources of emission spectra for atomic emission detectors in flow-injection analysis. *Journal of Analytical Chemistry*.

[21] Wilson C. G., Gianchandani Y. B. (2002). Spectral detection of metal contaminants in water using an on-chip microglow discharge. *IEEE Transactions on Electron Devices*.

[22] Mitra B., Wilson C. G., Que L., Selvaganapathy P., Gianchandani Y. B. (2006). Microfluidic discharge-based optical sources for detection of biochemicals. *Lab on a Chip *.

[23] Greda K., Swiderski K., Jamroz P., Pohl P. (2016). Flowing liquid anode atmospheric pressure glow discharge as an excitation source for optical emission spectrometry with the improved detectability of Ag, Cd, Hg, Pb, Tl, and Zn. *Analytical Chemistry*.

[24] Zhang Z., Wang Z., Li Q., Zou H., Shi Y. (2014). Determination of trace heavy metals in environmental and biological samples by solution cathode glow discharge-atomic emission spectrometry and addition of ionic surfactants for improved sensitivity. *Talanta*.

[25] Greda K., Jamroz P., Dzimitrowicz A., Pohl P. (2014). Direct elemental analysis of honeys by atmospheric pressure glow discharge generated in contact with a flowing liquid cathode. *Journal of Analytical Atomic Spectrometry*.

[26] Shekhar R. (2012). Improvement of sensitivity of electrolyte cathode discharge atomic emission spectrometry (ELCAD-AES) for mercury using acetic acid medium. *Talanta*.

[27] Wang Z., Gai R., Zhou L., Zhang Z. (2014). Design modification of a solution-cathode glow discharge-atomic emission spectrometer for the determination of trace metals in titanium dioxide. *Journal of Analytical Atomic Spectrometry*.

[28] Manjusha R., Reddy M. A., Shekhar R., Kumar S. J. (2014). Determination of cadmium in Zircaloys by electrolyte cathode discharge atomic emission spectrometry (ELCAD-AES). *Analytical Methods*.

[29] Manjusha R., Reddy M. A., Shekhar R., Jaikumar S. (2013). Determination of major to trace level elements in Zircaloys by electrolyte cathode discharge atomic emission spectrometry using formic acid. *Journal of Analytical Atomic Spectrometry*.

[30] Yu J., Yang S., Sun D. (2016). Simultaneously determination of multi metal elements in water samples by liquid cathode glow discharge-atomic emission spectrometry. *Microchemical Journal*.

[31] Yu J., Yang S., Lu Q. (2017). Evaluation of liquid cathode glow discharge-atomic emission spectrometry for determination of copper and lead in ores samples. *Talanta*.

[32] Jamroz P., Greda K., Pohl P. (2012). Development of direct-current, atmospheric-pressure, glow discharges generated in contact with flowing electrolyte solutions for elemental analysis by optical emission spectrometry. *TrAC Trends in Analytical Chemistry*.

[33] Liu Y., Sun B., Wang L., Wang D. (2012). Characteristics of light emission and radicals formed by contact glow discharge electrolysis of an aqueous solution. *Plasma Chemistry and Plasma Processing*.

[34] Zhu Z., Huang C., He Q. (2013). On line vapor generation of osmium based on solution cathode glow discharge for the determination by ICP-OES. *Talanta*.

[35] Mezei P., Cserfalvi T., Kim H. J., Mottaleb M. A. (2001). The influence of chlorine on the intensity of metal atomic lines emitted by an electrolyte cathode atmospheric glow discharge. *Analyst*.

[36] Webb M. R., Andrade F. J., Hieftje G. M. (2007). Use of electrolyte cathode glow discharge (ELCAD) for the analysis of complex mixtures. *Journal of Analytical Atomic Spectrometry*.

[37] Pereyra M. T., Lista A. G., Fernández Band B. S. (2013). Quantification of uncertainty in mercury wastewater analysis at different concentration levels and using information from proficiency test with a limited number of participants. *Talanta*.

[38] Padma Shri T. K., Sriraam N. (2017). Comparison of *t*-test ranking with PCA and SEPCOR feature selection for wake and stage 1 sleep pattern recognition in multichannel electroencephalograms. *Biomedical Signal Processing and Control*.

[39] Yu J., Zhang X., Lu Q. (2017). Determination of calcium and zinc in gluconates oral solution and blood samples by liquid cathode glow discharge-atomic emission spectrometry. *Talanta*.

